# Supplementary figures and images for: Establishment of a ferroptosis-related gene signature for prognosis in lung adenocarcinoma patients
Source: PeerJ. 2021 Aug 6;9:e11931. doi: 10.7717/peerj.11931 (PMC8351575; doi:10.7717/peerj.11931)

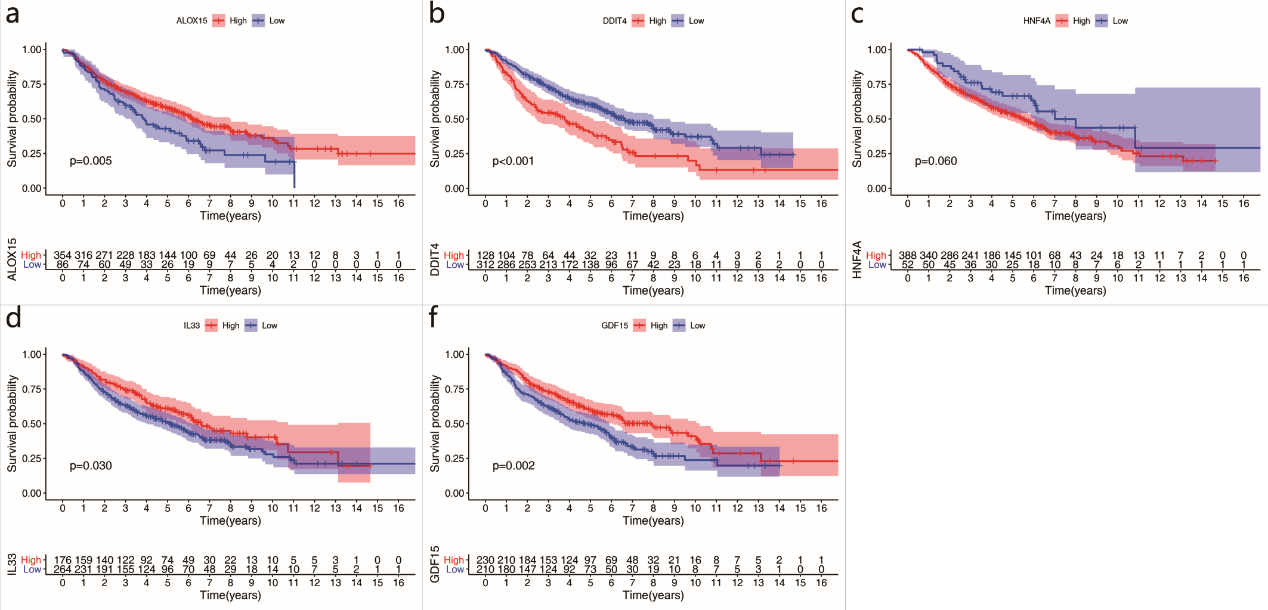

Supplement: Supplemental Information 1 [file peerj-09-11931-s001.png]

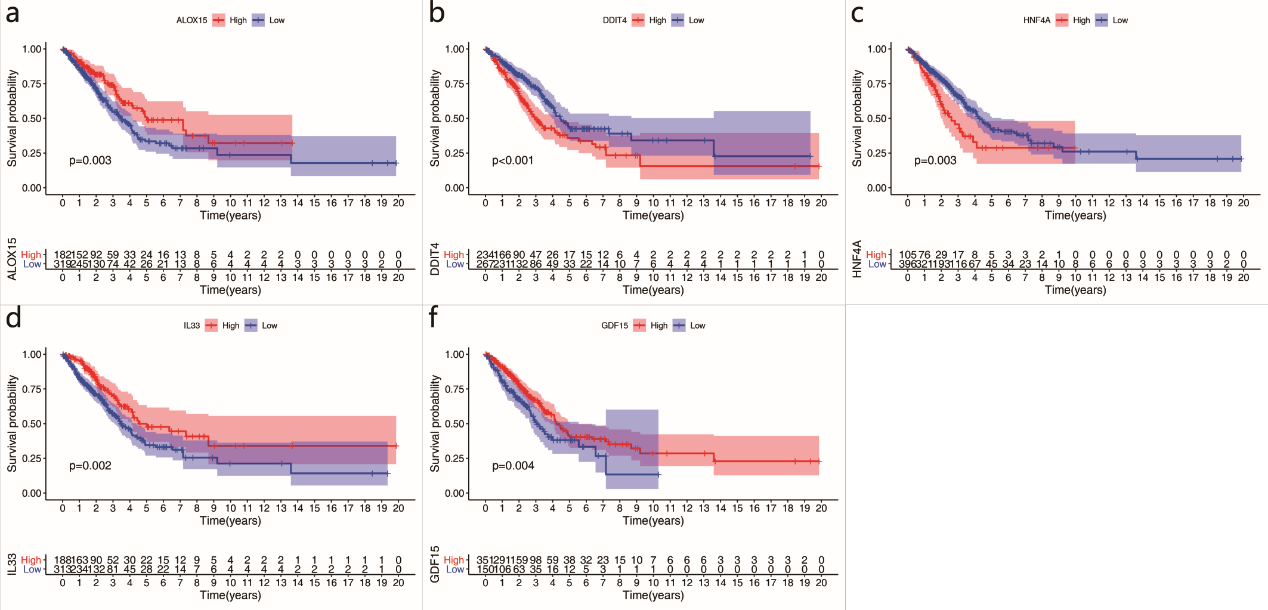

Supplement: Supplemental Information 2 [file peerj-09-11931-s002.png]
